# Spontaneous brachiocephalic artery dissection: a case report

**DOI:** 10.1093/jscr/rjaf040

**Published:** 2025-02-05

**Authors:** Lewis William Murray

**Affiliations:** Department of Cardiothoracic Surgery, Flinders Medical Centre, Adelaide, South Australia

**Keywords:** spontaneous, brachiocephalic, dissection

## Abstract

Spontaneous dissection of the brachiocephalic artery is incredibly rare event with only six reported cases in the medical literature. These dissections are associated with significant morbidity and mortality. Given the rarity, there is currently no clear consensus on the management and this present a significant challenge to clinicians. This case reports the successful management of a spontaneous brachiocephalic dissection in a 63-year-old male with pre-existing coronary disease.

## Introduction

An arterial dissection arises from a tear in the intimal layer of the arterial wall which allows blood to flow into the intraluminal space, and this separation results in the formation of a true and false lumen [1]. The expansion of the intramural haematoma can cause stenosis and/or obstruction of the true lumen due to clot extension or produce distal obstructions which are secondary to thromboembolism [2]. Common sites for dissection include the aorta, coronary arteries, cervical arteries, and the vertebral arteries [2]. Clinicians are likely to be familiar with sites; however, there are rarer sites that clinicians are less likely to have encountered. Arterial dissections that arise and are confined to the brachiocephalic artery is an extremely rare condition with only a small number reported in the literature [3].

We present a case of a spontaneous brachiocephalic artery dissection that presented with chest pain and without neurological symptoms.

## Case report

A 63-year-old man presented to our institution with sudden onset central chest pain that radiated from the sternum through to his back. The patient has a medical history of an inferolateral myocardial infarction in 2004 with a known complete occlusion of the distal right coronary artery, hypertension, hyperlipidaemia, and grade III chronic renal failure. His onset of symptoms began whilst the patient was at rest in bed and was described the chest pain as being similar in nature to that of a prior myocardial infarction. A paramedic crew attended his residence and administered sublingual glyceryl trinitrate (GTN) spray; however, this provided minimal reduction in the patient’s symptoms. Given his symptoms and his known history of ischaemic heart disease, he was transferred to the emergency department for further assessment and treatment.

**Figure 1:**
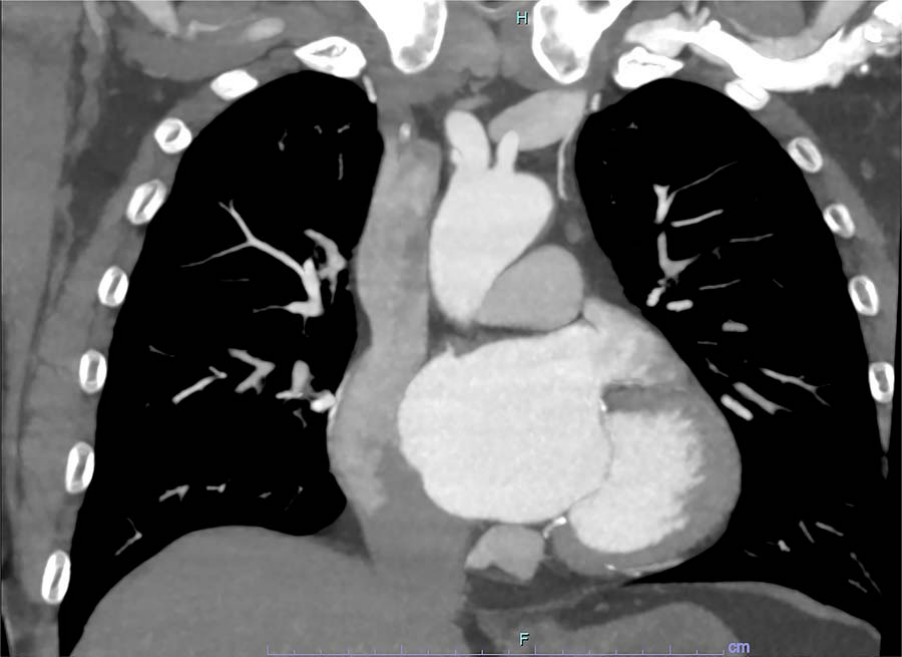
Coronal slice from CT aortogram demonstrating the origin of the dissection flap.

**Figure 2:**
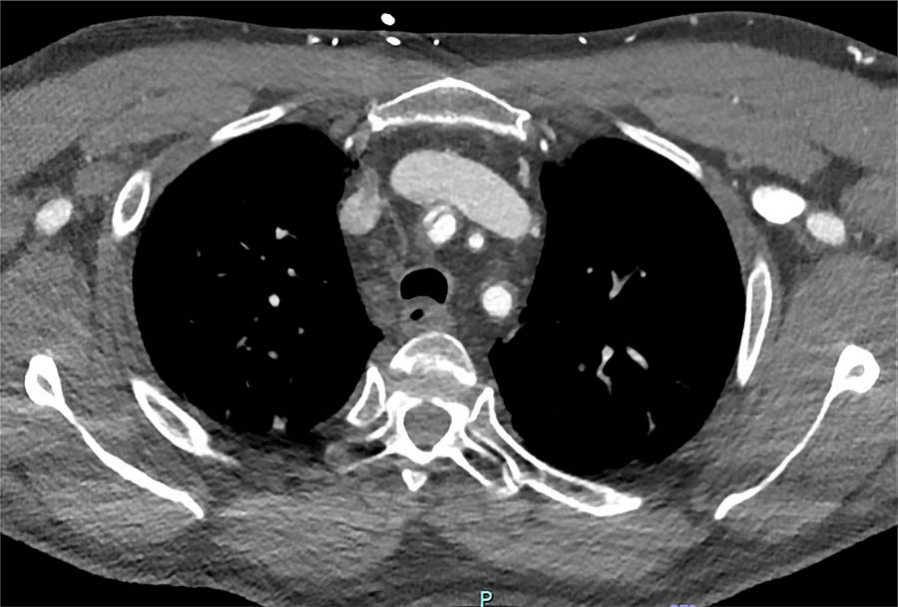
Axial slice from CT aortogram demonstrating the proximal aspect of the dissection flap.

**Figure 3:**
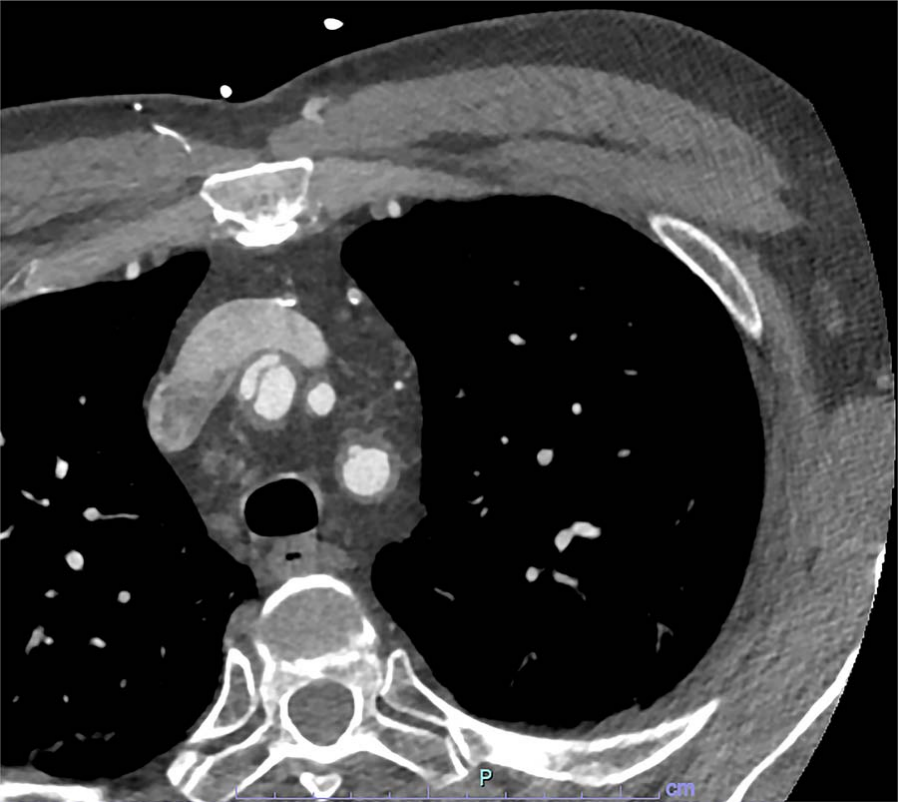
Axial slice from CT aortogram in greater detail of the dissection flap.

On arrive to the emergency department, the patient was found to be hypertensive with a blood pressure of 220/120 mmHg. Physical examination of the patient revealed no significant clinical findings. Serial troponins with corresponding ECG’s were undertaken which demonstrated no new evidence of myocardial ischaemia. Based upon the patient’s symptomatology, he underwent a CT aortogram which demonstrated a dissection arising from the proximal brachiocephalic artery. No dissection was noted in the ascending aorta, and there was no evidence of the brachiocephalic artery dissection extending into the subclavian or carotid arteries (Figs 1–3).

The patient was loaded with aspirin and commenced on both intravenous labetalol and GTN infusions to maintain strict blood pressure control. The patient was transferred to the intensive care unit for monitoring and careful conversion from intravenous to oral antihypertensive medications which was achieved over a 24 hour period. A CT brain was undertaken given the risk of intracranial thromboembolic events secondary to the dissection location. Thankfully there was no radiological evidence of acute or chronic stroke. A routine transthoracic echocardiogram was also undertaken which demonstrated normal left ventricular function and no valvular pathology.

Given the rarity of the patient’s pathology, their case was discussed at a cardiology multidisciplinary meeting to provide the patient with a consensus medical opinion. The recommendations were therapeutic anticoagulation with Apixaban given the risk of stroke and ongoing radiological surveillance. If there was radiological evidence of progression, then surgical intervention should be undertaken.

The patient underwent a follow up CT aortogram 3 months after initial presentation which demonstrated stable disease. The patient reported no new symptoms of any kind and was planned for further follow-up with repeat imaging in another 3 months’ time.

## Discussion

The common causes of innominate artery dissection include blunt or penetrating traumatic chest injuries, aortic dissection, iatrogenic arterial injuries, and secondary to connective tissues disorders [4]. Spontaneous brachiocephalic artery dissections are rare events and to date there have only been six reported cases of spontaneous dissection [3, 5–9].

The standard of care for the treatment of an ascending aortic dissection is urgent surgical repair [10]. However, unlike aortic dissections, there is currently no treatment guidelines for isolated brachiocephalic artery dissections given their rarity [5, 7]. Typically, supra-aortic aneurysms which are uncomplicated can be initially treated non-operatively [8]. Current treatment options reported in the medical literature can broadly defined as being either medical therapies that focus on blood pressure control and anticoagulation to prevent neurological injury or surgical intervention. Neurological symptoms as the presenting compliant occurred in three of the previously reported six cases [3, 5, 7].

There are two cases which reported propagation of the dissection to involve the ascending aorta, at which point then underwent surgery [5, 6]. One of these cases the patient unfortunately died, and these cases highlight the risk of propagation to an aortic dissection and the associated mortality [5]. For comparison, patients with residual dissections of the brachiocephalic artery following repair of an ascending aortic dissection have been reported as occurring in up to 30% of cases [11]. These residual brachiocephalic artery dissections pose a risk of further propagation as well as carry a risk of stroke which highlights the need for careful consideration of when to commence anticoagulation therapy [9].
